# Endosomal escape mechanisms of extracellular vesicle-based drug carriers: lessons for lipid nanoparticle design

**DOI:** 10.20517/evcna.2024.19

**Published:** 2024-07-05

**Authors:** Lasse Hagedorn, David C. Jürgens, Olivia M. Merkel, Benjamin Winkeljann

**Affiliations:** ^1^Department of Pharmacy, Ludwig-Maximilians-Universität München, München 81377, Germany.; ^2^Center for NanoScience (CeNS), Ludwig-Maximilians-Universität München, München 80799, Germany.; ^3^RNhale GmbH, München 81371, Germany.

**Keywords:** RNA therapeutics, drug delivery, lipid nanoparticles, membrane fusion, endosomal escape, extracellular vesicles

## Abstract

The rise of biologics and RNA-based therapies challenges the limitations of traditional drug treatments. However, these potent new classes of therapeutics require effective delivery systems to reach their full potential. Lipid nanoparticles (LNPs) have emerged as a promising solution for RNA delivery, but endosomal entrapment remains a critical barrier. In contrast, natural extracellular vesicles (EVs) possess innate mechanisms to overcome endosomal degradation, demonstrating superior endosomal escape (EE) compared to conventional LNPs. This mini review explores the challenges of EE for lipid nanoparticle-based drug delivery, and offers insights into EV escape mechanisms to advance LNP design for RNA therapeutics. We compare the natural EE strategies of EVs with those used in LNPs and highlight contemporary LNP design approaches. By understanding the mechanisms of EE, we will be able to develop more effective drug delivery vehicles, enhancing the delivery and efficacy of RNA-based therapies.

## INTRODUCTION

RNA-based drugs, with precisely controllable mechanisms of action and the ability to target various diseases at their root, offer substantial potential as therapeutics. This strategy could address untreatable conditions, revolutionizing treatments with potentially fewer side effects and greater efficacy compared to current options^[1,2]^

However, the therapeutic use of RNA faces significant challenges. Nucleic acids, especially RNAs, are susceptible to rapid degradation by enzymes (nucleases) or elimination via renal filtration without further protection strategy^[3]^. Moreover, the relatively large size of RNA molecules (around 22 to 25 base pairs for siRNA and over 1000 nucleotides for mRNA) and their polyanionic nature hinder passive diffusion across biological membranes into the cell’s cytosol - the primary site of action for RNA medicines^[3]^. To overcome these hurdles, the development of specialized drug delivery systems is essential.

Various approaches for delivering RNA-based therapeutics are under exploration, each offering distinct advantages and potential limitations. Biodegradable polymers, for example, can be designed to encapsulate and protect nucleic acids, offering controlled release properties^[4]^. Highly branched cationic molecules such as dendrimers can very efficiently complex with RNA molecules and offer customizable surface groups^[5]^. Directly conjugating RNA to molecules like peptides, antibodies, or lipids can facilitate targeted delivery and enhance its uptake by respective cell types^[6,7]^.

Lipid nanoparticles (LNPs) have emerged as leading carriers for RNA therapeutics, offering protection and transport capabilities^[8]^. This progress has already translated into clinical success, with several LNP-based drugs gaining approval. Notable examples include Onpattro® (patisiran), the first FDA-approved siRNA drug for the treatment of hereditary transthyretin-mediated amyloidosis, and vaccines against COVID-19 based on mRNA technology^[9]^. Market projections indicate substantial growth, with the LNP-based RNA therapeutics market surpassing $50 billion by 2030^[10]^.

Nonetheless, a persistent bottleneck remains: endosomal escape (EE). After entering cells, LNPs, like many drug delivery vehicles, often become trapped within endosomes. The endosomal-lysosomal system is a dynamic cellular network responsible for sorting and processing internalized cargo. After uptake via endocytosis, most drug delivery vehicles enter early endosomes, the cell’s primary sorting station^[11]^. Within the EE, molecules can be recycled back to the plasma membrane or progress toward late endosomes^[12]^. The decision to recycle or continue into the lysosomal pathway is guided by factors such as the composition of membrane domains, pH gradients within the early endosome, and specific molecular tags like ubiquitin^[11-13]^. Late endosomes are characterized by a lower pH and often fuse with lysosomes - the cell’s dedicated degradation compartments. Lysosomes contain a potent collection of hydrolytic enzymes that operate optimally in an acidic environment^[12,14]^. This presents a major hurdle for LNPs and other drug delivery systems. To avoid degradation, they must orchestrate their escape from the endosome before encountering this harsh lysosomal environment. The development of escape strategies is crucial for ensuring the successful delivery of their therapeutic payload.

In seeking solutions to this challenge, we can draw inspiration from nature’s own communication system. Extracellular vesicles (EVs) are membrane-enclosed particles naturally secreted by a wide range of cells, serving as vital intercellular communication vehicles^[15]^. They encompass diverse subtypes, including exosomes, microvesicles/ectosomes, and apoptotic bodies. These subtypes differ in their origins within the cell. Their ability to package and transport diverse bioactive molecules like proteins, lipids, and nucleic acids across significant distances *in vivo* positions them as promising therapeutic delivery systems^[16]^. Notably, studies have demonstrated that the EE performance of certain EVs can be more than 10-fold higher than commercial lipid nanoparticles^[17-19]^. Understanding the underlying mechanisms stands to provide valuable blueprints for enhancing the design of synthetic drug delivery systems, enabling us to overcome a fundamental barrier to their clinical success.

## CONTEMPORARY STRATEGIES FOR DESIGNING LIPID NANOPARTICLES FOR EE

### Past and present of lipid nanoparticles in a nutshell

Liposomes composed of positively charged lipids were among the earliest medical-chemistry-based attempts to deliver RNA^[20]^. The net positive charge promoted interaction with negatively charged cell membranes, prevented aggregation of the nanoparticles, and facilitated RNA encapsulation. Once the liposomes had been taken up via endocytosis, this positive charge also proved beneficial for disrupting the endosomal membrane. Unfortunately, the persistent cationic character often induces both pro-inflammatory cytokines and type I interferon by TLR4 activation, thereby disrupting cellular membranes, not just endosomal, resulting in unwanted side effects^[21,22]^.

**Figure 1:**
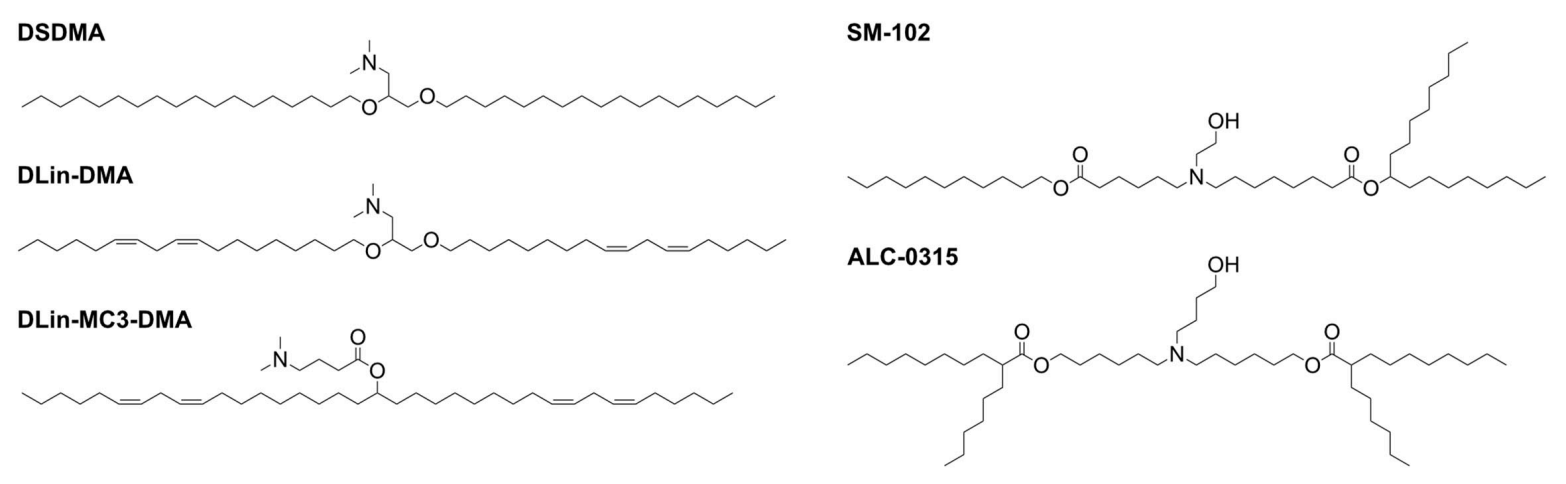
Ionizable lipids used in LNP formulations. Ionizable lipids used in clinical trials mostly contain head groups with pKa values below 7. Furthermore, the ratio between head group and tail group volume has been optimized to reach the optimal lipid per membrane area for sufficient endosomal escape. This resulted in different lipids with 2-4 alkyl tails with different degrees of saturation of the alkyl tails. LNP: Lipid nanoparticle.

To overcome this problem, researchers developed the concept of ionizable lipids. These lipids contain structural components with pKa values below 7 [Figure 1]. This ensures that the lipid is only positively charged under acidic conditions like those found within the endosomal compartment. This innovation allowed lipid-based nanocarriers to maintain a neutral charge at physiological pH, reducing cytotoxicity while still promoting EE through charge interaction with the endosomal membrane^[8]^.

The introduction of PEGylated lipids further advanced LNP technology^[23]^. The highly flexible structure, low intrinsic toxicity, and hydrophilic nature of PEG were shown to be beneficial and PEGylation significantly reduced aggregation during formulation and storage. By shielding charge and hindering opsonization, PEGylation also introduced stealth-like properties, increasing circulation half-life in the bloodstream by minimizing interactions with the immune system^[24]^. However, this effect of a stealth-like aqueous boundary layer formed by the PEG also results in less interaction with cell membranes and reduced uptake and EE^[25]^. Another downside is the lack of biodegradability of PEG. The necessity to balance the benefits of stealth properties of PEG against efficient uptake and endosomal release - known as the “PEG Dilemma”^[26]^ - became a crucial aspect of LNP design^[24]^. As a result, the search for alternatives to PEGylation is already underway, with polyoxazolines and polysarcosines being promising examples^[27,28]^.

Continued advancements in both lipid design and formulation processes have significantly improved LNP performance. The development of microfluidic and jet mixing techniques offered a way to generate highly reproducible nanoparticles on both small and industrial scales, with precise control over mixing procedures for optimal LNP formation^[29-31]^. These advancements culminated in a landmark achievement - the first FDA-approved RNAi therapeutic encapsulated in lipid nanoparticles in 2018^[9,32]^.

**Figure 2:**
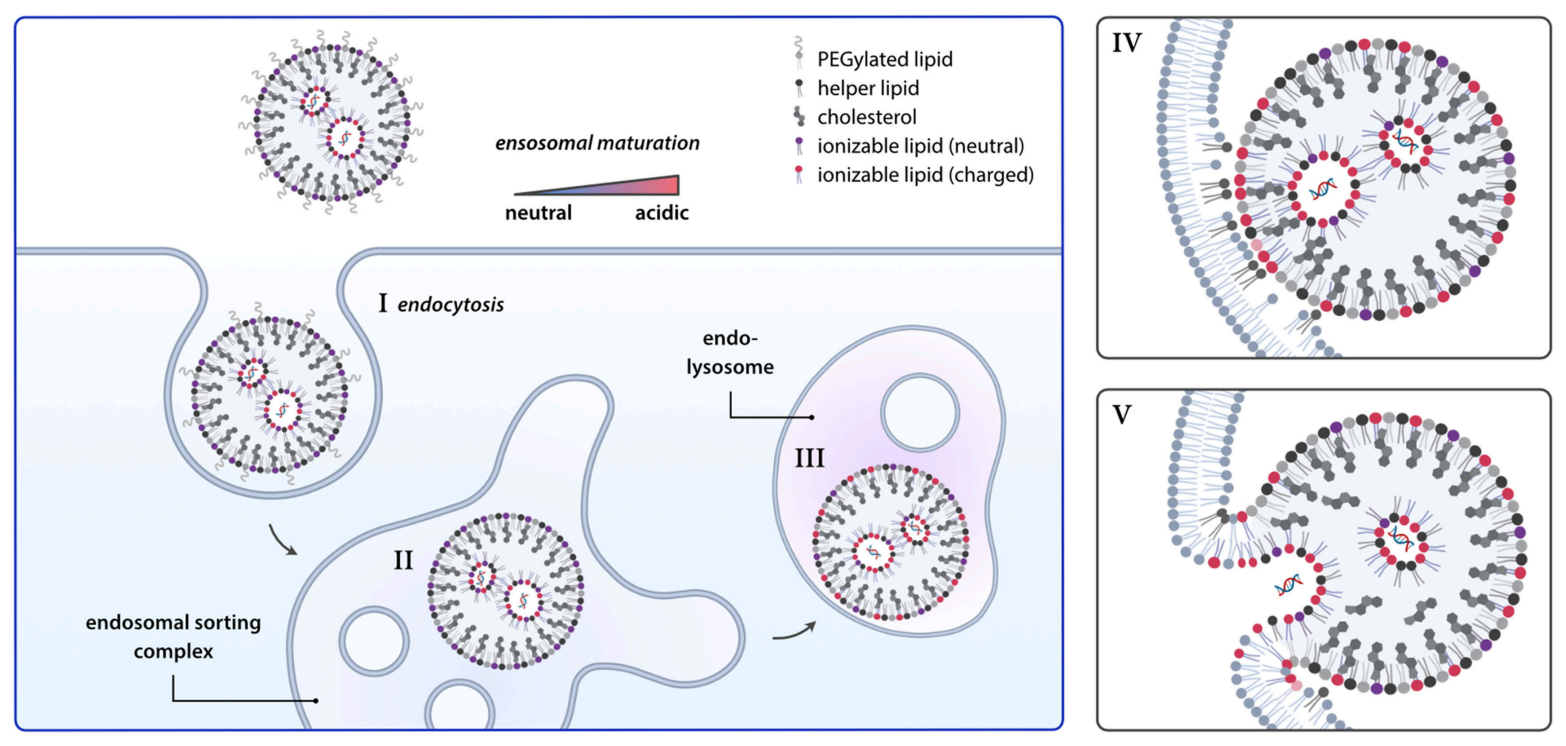
Endosomal escape of LNPs. Following endocytosis, LNPs follow the endo-lysosomal pathway. With the environment becoming increasingly acidic, ionizable lipids become protonated again. Now, the positively charged ionizable lipids can interact with negative membrane lipids to facilitate fusion with the endosomal membrane and release of the nucleic acid into the cytosol. Parts of this figure were created using Biorender. LNPs: Lipid nanoparticles.

Modern LNPs possess several key functions: they protect RNA payloads from nucleases and unwanted immune reactions, enable transport from the site of administration to the target tissue, and ideally, exhibit stealth-like properties to evade the immune system^[4]^. To achieve this, FDA-approved lipid nanoparticle-based nucleic acid therapeutics share a typical composition. Ionizable lipids facilitate RNA encapsulation and EE [Figure 2]. Phospholipids form the core structural component of the nanoparticle, while cholesterol-type lipids contribute to stability and enhance rigidity. Finally, PEGylated lipids provide a protective surface layer, improving circulation time.

### Mechanism of EE

The EE mechanism of LNPs is complex and not yet fully understood. This section aims to provide a comprehensive understanding of the proposed mechanisms and efforts to enhance the probability of EE. The need to understand and improve the EE is highlighted by findings that only 1%-2% of encapsulated siRNA formulated within commercially available LNPs entering an endosome are successfully released into the cytosol, while the majority are trafficked to the extracellular membrane^[33,34]^.

To improve EE, research has primarily focused on the role of ionizable lipids(ILs) in the composition of LNPs^[19]^. ILs are typically defined by their chemical structure, which features a small ionizable head group with a pKa between 6 and 7, and a nonpolar tail group that includes either saturated or unsaturated alkyl chains with varying degrees of branching [Figure 1]^[35]^. This specific pKa range allows the lipid to become positively charged during the RNA encapsulation process, facilitated by an aqueous acidic buffer, and then return to its unprotonated state following dialysis against neutral buffers, resulting in the final LNP formulation. Measurements conducted through Small Angle Neutron Scattering (SANS) and Small Angle X-ray Scattering (SAXS) have subsequently verified that the ionizable lipid is predominantly located within the LNP, in close proximity to the RNA^[36]^.

In the initial stages of investigating the chemical properties of ILs, the influence of alkyl chain saturation and pKa range was prioritized. It was discovered that DSDMA, characterized by fully saturated hydrophobic chains, exhibited the highest cellular uptake but the lowest transfection efficiency. In contrast, DLinDMA, which contains two double bonds, showed a knockdown efficiency of nearly 80%. To unravel the reasons behind the varying transfection efficiencies, comparisons using P31-NMR revealed that the phase transition temperature of the ILs decreases as the number of double bonds in the alkyl chain increases, which correlates with fusogenic activity due to the adaption of the lamellar phase (L) to an inverted hexagonal phase (H_II_). This trend becomes prominent with up to two double bonds, beyond which the differences become negligible^[37]^. Advances in IL design have concentrated on inducing the phase transition of the endosomal membrane bilayer to increase EE probability. This has been facilitated by altering the shape of the ILs. Traditional cylindrically shaped lipids tend to have a smaller membrane area per lipid as they can be packed more tightly. Therefore, these lipids are more likely to form the inner leaflet of a membrane, often described as lipids with intrinsic negative curvature. Compared to these cylindrical lipids, double bonds were introduced into ILs and the branching degree of the nonpolar tail groups was increased, thereby forming a more cone-like structure^[38]^. These newly designed ILs have a high membrane area per lipid, especially when interacting with negatively charged lipids from the inner side of the membrane of the endosome. As a result, these lipids with an intrinsic positive curvature can lead to phase transition of the lipid bilayer membrane of endosomes^[39,40]^. Such modifications have led to increased mRNA transfection efficiency compared with the Dlin-MC3 counterpart. Notable examples include SM-102, used in Moderna’s vaccine, and ALC-0315, featured in BioNTech/Pfizers’s mRNA vaccine^[41]^. For the next generation of ionizable lipids and lipid compositions, innovative strategies have been developed. By leveraging the knowledge of the hexagonal phase transition, greater branching can be utilized to enhance the design and function of these lipids. Additionally, the biocompatibility of ionizable lipids (ILs) can be increased by introducing biodegradable structural elements, such as disulfide bonds, which can be cleaved inside the cell^[42]^. A recent publication introduces another bottom-up approach, demonstrating that a liquid crystalline inverse hexagonal lipid phase within LNPs, directed by lipid composition and ratios, can enhance transfection efficiency compared to lamellar phases^[43]^.

The significance of phospholipids such as DOPE or DSPC and PEGylated lipids such as PEG-DMG in interfering with the EE is also important. DOPE can also promote the formation of the hexagonal phase transition of the endosomal membrane, while DSPC is currently used in approved LNP formulation by enhancing the overall stability of the LNP^[25]^. On the other hand, the percentage of PEGylated lipids, while essential for extending the circulation time and improving the biodistribution of LNPs, requires careful optimization as EE can be negatively influenced^[26]^. This balance between enhancing EE and preserving the nanoparticle’s stability and distribution highlights the complexity involved in the formulation of effective RNA delivery systems.

Another postulated mechanism for the EE is the proton sponge effect. This theory, primarily associated with polymeric-based mechanisms of EE, posits that upon their protonation, ionizable lipids induce an influx of chloride ions into the endosome, which can be controlled by adjusting the buffering capacity of the IL. This leads to osmotic swelling and, ultimately, the rupture of the endosomal membrane^[44]^.

The process of optimizing ILs also necessitates a careful evaluation of the target cells. Since mechanistic studies on EE are predominantly conducted *in vitro*, it is essential to acknowledge that the pathways of internalization and endosomal processes, along with pH levels, can differ significantly among various cell types. This variability underscores the complexity of accurately predicting and enhancing EE in a physiological context^[44,45]^.

It is important to emphasize that EE must be both effective and safe. Recent studies have shown a correlation between high EE efficiency and inflammation. This observation was shown to be linked to irreversible damage some of the highly potent ILs cause to the endosomes. Nevertheless, exceptions were also found: the ionizable lipid 4A3-SC8 achieves high mRNA expression while causing low inflammation, suggesting high EE efficiency can be achieved without permanently damaging the endosomal compartment. Furthermore, the study pinpoints that enhanced biodegradability is a desirable feature in the design of new lipids. This is a characteristic that could be attained by designing lipids inspired by the natural properties of extracellular vesicles^[46]^.

Lastly, mRNA has been detected in secreted exosomes following LNP internalization, which could then potentially be taken up by recipient cells^[47]^. This suggests a multifaceted mechanism of action, where not only the direct delivery of LNPs but also the subsequent intercellular communication through exosomes play critical roles in the effective transfection. This insight opens up additional avenues for research and optimization in the field of RNA delivery, highlighting the dynamic and interconnected nature of cellular processes involved in gene therapy.

## EXTRACELLULAR VESICLES IN DRUG DELIVERY

EVs encompass a diverse range of cell-secreted particles with promising potential as therapeutic delivery vehicles. One of the current biggest hurdles is the clear isolation and characterization of respective subtypes. EVs can broadly be defined by their size, with small EVs often marked by a diameter smaller than 200 nm and large EVs above 200 nm. Another approach is the categorization by their origin. For example, exosomes are small EVs that form within multivesicular bodies (MVBs) and are released when MVBs fuse with the cell membrane. In contrast, ectosomes are EVs of comparable size to exosomes but originate directly from the plasma membrane^[48]^. Due to their endosomal origin, exosomes might possess intrinsic mechanisms for EE, making them particularly interesting for drug delivery^[15]^. Microvesicles form through direct outward budding of the cell membrane, and their larger size may be advantageous for carrying greater amounts of therapeutic cargo^[16]^. Apoptotic bodies, arising from the process of programmed cell death, have shown promise for encapsulating and delivering complex biological molecules, including drugs, facilitating targeted and efficient therapeutic interventions^[49,50]^. It is important to consider that while there can be overlap in size or origin, a population of EVs may still contain multiple different EV subtypes. Depending on their cargo, these subtypes can have drastically different effects on their recipient cells^[48]^. Understanding the biogenesis is crucial for effectively utilizing their potential in drug delivery. Researchers are actively seeking specific markers to reliably distinguish between EV subtypes, which will further enhance their tailored use.

EVs offer several benefits for carrying drugs compared to synthetic or virus-based carriers. Their natural membrane structure provides biocompatibility and some degree of protection for the therapeutic cargo. The specific properties of different EV types may be exploited for tailored delivery strategies. Researchers are investigating various methods to load drugs into EVs. One strategy involves passive diffusion, where small, lipophilic drugs may naturally diffuse across the EV membrane and become encapsulated. Electroporation is another techniqu, in which electrical pulses are used to create temporary pores in the EV membrane, allowing for the loading of larger or less lipophilic therapeutic molecules such as nucleic acids^[51]^. A third approach involves genetically or metabolically modifying the cells that produce EVs. This can enable the cells to directly package specific therapeutic molecules into the EVs during their formation^[52]^.

The field of EVs as drug delivery systems holds great promise, yet no EV-based therapy has been approved by the FDA to date. However, some Phase 3 trials are currently in progress. One notable example is the EXTINGUISH ARDS trial (NCT05354141), which is evaluating the safety and efficacy of ExoFlo, a treatment derived from bone marrow mesenchymal stem cell extracellular vesicles, for moderate-to-severe acute respiratory distress syndrome from any cause. This trial, which started in 2022, is expected to be completed by August 2025. Most clinical trials are utilizing EVs as biomarkers. Along with these, there are several Phase I clinical trials using EVs as drug delivery systems carrying siRNA (NCT03608631), miRNA (NCT03384433) and mRNA (NCT05043181)^[53]^.

### EE mechanisms of EVs

**Figure 3:**
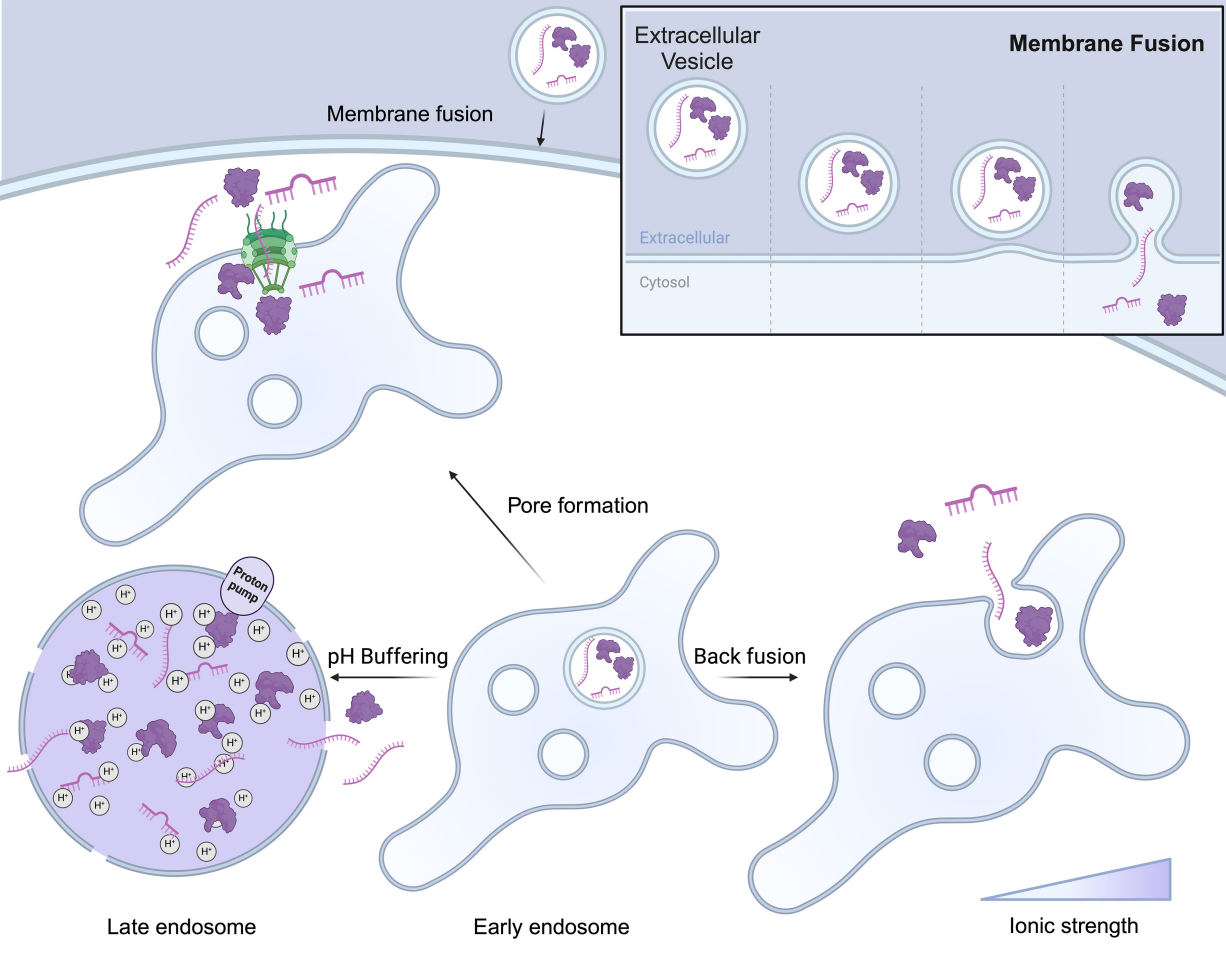
Escape mechanisms of extracellular vesicles. There are four proposed mechanisms for delivering EV cargo into the cytoplasm. The first mechanism involves the direct fusion of the EV with the cellular membrane, resulting in the complete release of the EV’s contents. The second mechanism, known as back fusion, occurs when the EV fuses with the endosomal membrane after being taken up via endocytosis. In the third mechanism, following endosomal uptake, EVs can induce pore formation, allowing the cargo to be transported into the cytoplasm. Finally, EVs can promote endosomal escape by buffering acidification and increasing the number of protons transported into the endosome, leading to membrane leakage through osmotic pressure caused by high ionic strength. This figure was created using Biorender. EV: Extracellular vesicle.

Understanding how EVs naturally achieve EE is crucial for designing effective drug delivery systems. Over the years, different mechanisms have been proposed and investigated that enable EV-based drug delivery systems to escape the endosomal entrapment [Figure 3]^[54]^.

#### Membrane fusion

EVs possess the remarkable ability to escape the endosomal compartment through direct membrane fusion with the endosomal membrane [Figure 3]. This allows EVs to release their therapeutic contents directly into the cell’s cytoplasm. The unique lipid and protein composition of the EV membrane plays a crucial role in enabling this process. Specific lipids, like ceramide, contribute to the formation of inward-budding vesicles by inducing curvature of the membrane. Additionally, cholesterol, an important component of MVBs, enriches the membranes of exosomes, facilitating exosome secretion^[55]^. Furthermore, both EV membranes and endosomal membranes contain specialized proteins that promote membrane fusion. These include SNARE proteins (SNAP receptors), which form complexes that bring membranes into close proximity, and Rab proteins, which regulate various stages of membrane trafficking and fusion^[54]^. Recent research identified syntaxin-4, SNAP-23, and VAMP-7 as the cognate SNAREs mediating MVB-PM fusion, crucial for exosome secretion in MCF-7 breast cancer cells^[56]^. Tetraspanins, a family of membrane proteins, have also been implicated in facilitating vesicle fusion events, with studies highlighting their role in cargo sorting and exosome secretion. The tetraspanin family members, including CD9, CD63, and CD81, are significantly involved in the regulation of cargo sorted for exosome secretion^[55]^. The mildly acidic environment within the endosome might play a role as well, potentially activating pH-sensitive proteins on the EV or endosomal membrane and triggering conformational changes that promote fusion. Research has provided valuable insights into the potential interactions that could underlie EV-mediated membrane fusion. For instance, studies suggest that the tetraspanin CD63 on the EV membrane might interact with specific lipids on the endosomal membrane to facilitate this process^[57]^.

#### Pore formation

EVs may utilize another fascinating EE mechanism: the ability to induce the formation of transient pores within the endosomal membrane. These pores can act as channels for the controlled release of EV cargo directly into the cytoplasm [Figure 3]^[58]^. Researchers are exploring specific lipids or proteins within the EV membrane as critical factors in triggering this pore-formation process. The presence of cone-shaped lipids within the EV membrane, such as phosphatidylethanolamine, is hypothesized to contribute to pore formation. Their insertion into the endosomal membrane could potentially disrupt its structure, leading to localized weaknesses and the creation of pores. Additionally, certain peptides, such as those derived from the influenza virus hemagglutinin-2 protein or the bacteriorhodopsin protein, can undergo pH-dependent conformational changes that enable them to insert into the endosomal membrane and disrupt its integrity^[59]^. Similarly, the incorporation of pH-sensitive peptides into the surface of extracellular vesicles is speculated to directly form pores, akin to mechanisms seen in microbial strategies for cell entry^[60]^. These specialized proteins might directly insert themselves into the endosomal membrane, creating channels for cargo release. It is proposed that lipids and proteins within the EV membrane work together to induce pore formation, with initial membrane destabilization by lipids setting the stage for protein insertion and subsequent pore formation^[60]^. Moreover, researchers identified importin ß1 within EVs through proteomic analysis. By comparing the nuclear delivery efficiency of labeled EV components with and without importazole, an inhibitor of ß1 importin, they have proven the importance of ß1 for the nuclear delivery of EVs from the endosome. These findings highlight the importance of different molecules within the EVs and how different EV compositions may influence their delivery efficiency depending on the cell type and target compartment, further highlighting the importance of identifying the subtypes of EVs^[61]^. Additionally, understanding the mechanisms of EV-mediated pore formation is an ongoing research area, crucial for elucidating the specific molecules involved and the detailed processes leading to the disruption of endosomal membrane integrity through pore formation^[16]^.

#### “Back-fusion”

EVs might exploit another unique mechanism to escape the endosomal compartment, known as “back-fusion”. Multivesicular bodies (MVBs) are specialized compartments within the endosome that contain smaller intraluminal vesicles (ILVs). While it is established that MVBs can fuse with the cell’s plasma membrane to release ILVs as exosomes, it is also theorized that MVBs might undergo a reverse process, or “back-fusion”, with the outer endosomal membrane. This would enable the release of any internalized EVs trapped within the MVB directly into the cytoplasm, thereby evading lysosomal degradation [Figure 3]^[62]^. Similar to other membrane fusion events, the “back-fusion” of MVBs with the endosomal membrane likely involves specialized proteins such as SNAREs, which are known to facilitate the proximity of membranes for fusion, and Rab proteins, which are crucial in regulating the stages of membrane trafficking and fusion^[18,63]^. The feasibility of “back-fusion” as an EE mechanism may depend on various factors, including the specific lipid and protein composition of both the MVB and the endosomal membranes. Additionally, the nature of the cargo within the EV might play a role in its sorting into MVBs destined for “back-fusion” rather than lysosomal degradation. Supporting evidence for the “back-fusion” hypothesis includes research on exosomes derived from hypoxia-conditioned mesenchymal stem cells, which suggests that these exosomes can enhance intercellular communication with injured heart cells, potentially contributing to repair mechanisms^[62]^.

#### pH buffering

Endosomes maintain a mildly acidic internal environment due to the action of proton pumps. This acidity plays a critical role in the sorting and degradation processes within the endosome. Interestingly, extracellular vesicles (EVs) might possess an intrinsic ability to counter this acidification through pH buffering. Components within EVs, potentially specific proteins and membrane lipids, may have buffering capacities that minimize drastic pH shifts. By preventing significant acidification, these EVs could protect their cargo and themselves from degradation by pH-sensitive lysosomal enzymes. Researchers are actively investigating the precise mechanisms behind EV pH buffering. Studies suggest that certain EV proteins might play a role in capturing or transporting protons, thus modulating the endosomal environment. Recent research has revealed that treating cells with bafilomycin A1, an inhibitor of the endosomal acidification process, leads to decreased transfection rates. This suggests that the EE mechanism does not directly result from the pH buffering effect. The theory is that buffering the acidification in the endosome causes the proton pumps responsible for acidification to increase their activity, transporting more ions into the endosome. This rise in ionic strength increases the osmotic pressure, which may cause the endosome to rupture [Figure 3]. To investigate this trend, researchers developed GFP-conjugated CD63-containing EVs from HEK293T cells and delivered them to cells containing anti-GFP nanobodies fused with mCherry. The presence of red and green co-spots indicated that CD63 was released into the cytosol. However, when endosome acidification was hindered with bafilomycin A1, there was a dramatic decrease in these co-spots compared to untreated cells^[61]^. Additionally, the unique lipid composition of the EV membrane could contribute to its buffering capacity^[64]^.

The relative importance of these EE mechanisms is likely context-dependent, influenced by factors such as the EV subtype, its cargo, and the target cell type. Continuous research is crucial to fully understand these complex processes and their implications for the design of next-generation EV-based drug delivery systems.

## LESSONS LEARNED - HOW NATURE’S WAY MIGHT BE UTILIZED TO BOOST THE EE PERFORMANCE OF THE NEXT GENERATION OF LNPs

The EE rate of commercially available LNPs is limited to 1%-2%^[34]^, whereas for exosomes, rates higher than 20%^[18]^ are reported in the literature. Increased cytosolic release could hold therapeutic significance across various fields, potentially reducing the need for excipients in RNA-based therapies such as vaccinations or cancer treatments. A higher concentration of available RNA in the cytosol could lead to enhanced treatment efficacy, raising the question of what LNPs could learn and adapt from EVs.

We have learned that the rational design of ionizable lipids is crucial. Specifically, an increased branching degree of the ionizable lipid tail can enhance the formation of a hexagonal phase with the endosomal membrane, facilitating EE and subsequent RNA release into the cytosol^[38]^. At this point, where hundreds of ILs have been screened to improve the current state of the art, the question arises whether the optimization of the chemical structure, including the optimal pKa and steric shape, has already reached its limits. To further explore opportunities to enhance treatment possibilities, a closer look at EVs is crucial due to their exceptional EE efficiency.

Could we, for example, leverage EV-associated pathways within the endosomes, such as the postulated tetraspanin, to target and control the fate of lipid nanoparticles or polymeric nanocarriers? With research still in its early stages, detailed mechanistic studies are essential to deepen our understanding of these processes. Gaining insights into how EVs naturally modulate their surroundings within the endosome could provide critical knowledge for developing bio-inspired LNPs. The inclusion of pH-buffering components or structural modifications in the lipid chemistry may safeguard sensitive therapeutic agents and enhance EE efficiency. While buffering-related mechanisms have been heavily investigated for polymeric-based carriers, there is a noticeable gap in investigations concerning LNPs^[65]^.

A survey conducted in 2019 among experts in the field of EVs showed a high level of agreement that both proteins displayed on EV surfaces and those present in the endosomal compartment are highly important for the interactions of EVs with their target cells. In addition, they pointed out that by manipulating the surface features of EVs, it is possible to manipulate the fate of EVs taken up by endocytosis^[66]^. Based on this, the attempt to adjust lipid composition within the LNP and therefore the LNP shell is very coherent. Most of this research nowadays focuses on biodistribution and uptake into specific cells^[67]^. Focusing on the protein corona and how to modify the LNPs to accumulate proteins favorable for EE may bring new strategies. Even preloading the LNPs with a protein corona is an attempt already tried in literature, but mostly for targeting^[68]^. Therefore, designing LNPs with a protein corona assembled of proteins could be capable of boosting EE, but it needs to have proteins with high affinity to the LNPs surface, while still not preventing uptake and biodistribution.

Another factor that can be utilized to increase delivery efficiency is the chemistry of RNA. For siRNA, chemical modifications to the ribose, phosphate, or base can enhance stability against nucleases or potency^[69]^. A notable example is the approved drug Vutrisiran for the treatment of hereditary transthyretin-mediated (hATTR) amyloidosis. By attaching the targeting ligand GalNAc (targeting the asialoglycoprotein receptor located on the extracellular membrane of hepatocytes) to the highly stabilized siRNA, it was possible to bypass the need for a delivery vector. Vutrisiran offers reduced dosing frequencies, necessitating administration only every three months^[70]^. This approach capitalizes on minimal EE, with residual RNA being stored in endosomes for a gradual release^[71]^. To further this approach, modified nucleic acids are encapsulated in LNPs, which either form a depot of nucleic acid in the endosome or promote EE. This could be achieved by conjugating ligands that promote EE in EVs, on the RNA. As a result, a deeper understanding of these proteins, for example, tetraspanins, their subtypes, and binding domains, is needed. However, it remains uncertain whether such treatment strategies are viable for longer RNA molecules like mRNA or saRNAs, especially in scenarios requiring high cytosolic RNA availability.

While the pharmacodynamics of LNPs have been extensively studied, their pharmacokinetics require further investigation^[61]^. Concerns also arise regarding their toxicological profile^[60]^. Unlike extracellular vesicles, which are composed of naturally occurring components, LNPs are artificial vectors. Therefore, the biodegradability and immunogenicity of each new component must be assessed. Incorporating naturally occurring structures from EVs into LNPs may reduce the risk of immune stimulation and bioaccumulation, potentially diminishing the need for extensive pharmacokinetic studies. Despite efforts to improve this aspect, clinical validation has not yet been achieved.

For EVs, the reproducible and reliable characterization, isolation and production of the final drug carrier is still a major hurdle, as shown by the clinical trial NCT03079401. Here, they managed to surpass the primary endpoint of phase 3 of the clinical trial, but did not get approval from the FDA because of the unreliable measurement of the final product’s biological activity^[72]^. Compared to the biogenesis and production of therapeutic EVs, LNPs are well-defined and reproducible drug carriers. Therefore, combining EVs and LNP to eliminate their downsides and push their strength is a promising attempt.

Lastly, translating these discoveries into *in vivo* studies or clinical applications, particularly in human subjects, presents considerable challenges. Considering the effect of preincubation of lipid nanoparticles in serum on their uptake and delivery efficiency, the absence of serum proteins in most *in vitro* experiments emphasizes this challenge^[61]^. No direct *in vitro* to *in vivo* correlation has been established for LNPs nor for EVs, evidenced by a low mouse-to-human correlation^[48,73,74]^. This discrepancy raises critical questions about the efficacy of study designs for both EVs and LNPs and whether they can inform the rational design of compositions to improve EE and future treatment possibilities.

## DECLARATIONS

### Copyright

© The Author(s) 2024.

## References

[1] Roberts TC, Langer R, Wood MJA (2020). Advances in oligonucleotide drug delivery. Nat Rev Drug Discov.

[2] Damase TR, Sukhovershin R, Boada C, Taraballi F, Pettigrew RI, Cooke JP (2021). The limitless future of RNA therapeutics. Front Bioeng Biotechnol.

[3] Dowdy SF (2017). Overcoming cellular barriers for RNA therapeutics. Nat Biotechnol.

[4] Mitchell MJ, Billingsley MM, Haley RM, Wechsler ME, Peppas NA, Langer R (2021). Engineering precision nanoparticles for drug delivery. Nat Rev Drug Discov.

[5] Kesharwani P, Banerjee S, Gupta U (2015). PAMAM dendrimers as promising nanocarriers for RNAi therapeutics. Mater Today.

[6] Cao W, Li R, Pei X (2022). Antibody-siRNA conjugates (ARC): emerging siRNA drug formulation. Med Drug Discov.

[7] Chernikov IV, Vlassov VV, Chernolovskaya EL (2019). Current development of siRNA bioconjugates: from research to the clinic. Front Pharmacol.

[8] Cullis PR, Hope MJ (2017). Lipid nanoparticle systems for enabling gene therapies. Mol Ther.

[9] Adams D, Gonzalez-Duarte A, O'Riordan WD (2018). Patisiran, an RNAi therapeutic, for hereditary transthyretin amyloidosis. N Engl J Med.

[10] https://www.bio.org/clinical-development-success-rates-and-contributing-factors-2011-2020.

[11] Huotari J, Helenius A (2011). Endosome maturation. EMBO J.

[12] Jovic M, Sharma M, Rahajeng J, Caplan S (2010). The early endosome: a busy sorting station for proteins at the crossroads. Histol Histopathol.

[13] Mohrmann K, Gerez L, Oorschot V, Klumperman J, van der Sluijs P (2002). Rab4 function in membrane recycling from early endosomes depends on a membrane to cytoplasm cycle. J Biol Chem.

[14] Scott CC, Vacca F, Gruenberg J (2014). Endosome maturation, transport and functions. Semin Cell Dev Biol.

[15] Kalluri R, LeBleu VS (2020). The biology, function, and biomedical applications of exosomes. Science.

[16] (2018). Niel G, D’Angelo G, Raposo G. Shedding light on the cell biology of extracellular vesicles. Nat Rev Mol Cell Biol.

[17] Hashemi A, Ezati M, Nasr MP, Zumberg I, Provaznik V (2024). Extracellular vesicles and hydrogels: an innovative approach to tissue regeneration. ACS Omega.

[18] Joshi BS, de Beer MA, Giepmans BNG, Zuhorn IS (2020). Endocytosis of extracellular vesicles and release of their cargo from endosomes. ACS Nano.

[19] Schlich M, Palomba R, Costabile G (2021). Cytosolic delivery of nucleic acids: the case of ionizable lipid nanoparticles. Bioeng Transl Med.

[20] Malone RW, Felgner PL, and Verma IM (1989). Cationic liposome-mediated RNA transfection. Proc Natl Acad Sci U S A.

[21] Syama K, Jakubek ZJ, Chen S, Zaifman J, Tam YYC, Zou S (2022). Development of lipid nanoparticles and liposomes reference materials (II): cytotoxic profiles. Sci Rep.

[22] Lv H, Zhang S, Wang B, Cui S, Yan J (2006). Toxicity of cationic lipids and cationic polymers in gene delivery. J Control Release.

[23] Mui BL, Tam YK, Jayaraman M (2013). Influence of polyethylene glycol lipid desorption rates on pharmacokinetics and pharmacodynamics of siRNA lipid nanoparticles. Mol Ther Nucleic Acids.

[24] Knop K, Hoogenboom R, Fischer D, Schubert US (2010). Poly(ethylene glycol) in drug delivery: pros and cons as well as potential alternatives. Angew Chem Int Ed Engl.

[25] (2022). Albertsen C, Kulkarni JA, Witzigmann D, Lind M, Petersson K, Simonsen JB. The role of lipid components in lipid nanoparticles for vaccines and gene therapy. Adv Drug Deliv Rev.

[26] Hatakeyama H, Akita H, Harashima H (2013). The polyethyleneglycol dilemma: advantage and disadvantage of PEGylation of liposomes for systemic genes and nucleic acids delivery to tumors. Biol Pharm Bull.

[27] Nogueira SS, Schlegel A, Maxeiner K (2020). Polysarcosine-functionalized lipid nanoparticles for therapeutic mRNA delivery. ACS Appl Nano Mater.

[28] Sanchez AJDS, Loughrey D, Echeverri ES (2024). Substituting poly(ethylene glycol) lipids with poly(2-ethyl-2-oxazoline) lipids improves lipid nanoparticle repeat dosing. Adv Healthc Mater.

[29] Shepherd SJ, Issadore D, Mitchell MJ (2021). Microfluidic formulation of nanoparticles for biomedical applications. Biomaterials.

[30] Jürgens DC, Deßloch L, Porras-gonzalez D (2023). Lab-scale siRNA and mRNA LNP manufacturing by various microfluidic mixing techniques - an evaluation of particle properties and efficiency. OpenNano.

[31] O’Brien Laramy MN, Costa AP, Cebrero YM (2023). Process robustness in lipid nanoparticle production: a comparison of microfluidic and turbulent jet mixing. Mol Pharm.

[32] Akinc A, Maier MA, Manoharan M (2019). The Onpattro story and the clinical translation of nanomedicines containing nucleic acid-based drugs. Nat Nanotechnol.

[33] Sahay G, Querbes W, Alabi C (2013). Efficiency of siRNA delivery by lipid nanoparticles is limited by endocytic recycling. Nat Biotechnol.

[34] Gilleron J, Querbes W, Zeigerer A (2013). Image-based analysis of lipid nanoparticle-mediated siRNA delivery, intracellular trafficking and endosomal escape. Nat Biotechnol.

[35] Sharma R, Lee JS, Bettencourt RC, Xiao C, Konieczny SF, Won YY (2008). Effects of the incorporation of a hydrophobic middle block into a PEG-polycation diblock copolymer on the physicochemical and cell interaction properties of the polymer-DNA complexes. Biomacromolecules.

[36] Schoenmaker L, Witzigmann D, Kulkarni JA (2021). mRNA-lipid nanoparticle COVID-19 vaccines: structure and stability. Int J Pharm.

[37] Heyes J, Palmer L, Bremner K, MacLachlan I (2005). Cationic lipid saturation influences intracellular delivery of encapsulated nucleic acids. J Control Release.

[38] Xu Y, Golubovic A, Xu S, Pan A, Li B (2023). Rational design and combinatorial chemistry of ionizable lipids for RNA delivery. J Mater Chem B.

[39] Philipp J, Dabkowska A, Reiser A (2023). pH-dependent structural transitions in cationic ionizable lipid mesophases are critical for lipid nanoparticle function. Proc Natl Acad Sci U S A.

[40] Pabst G, Keller S (2024). Exploring membrane asymmetry and its effects on membrane proteins. Trends Biochem Sci.

[41] Escalona-Rayo O, Zeng Y, Knol RA (2023). *In vitro* and *in vivo* evaluation of clinically-approved ionizable cationic lipids shows divergent results between mRNA transfection and vaccine efficacy. Biomed Pharmacother.

[42] Chen Z, Tian Y, Yang J (2023). Modular design of biodegradable ionizable lipids for improved mRNA delivery and precise cancer metastasis delineation in vivo. J Am Chem Soc.

[43] Pattipeiluhu R, Zeng Y, Hendrix MMRM, Voets IK, Kros A, Sharp TH (2024). Liquid crystalline inverted lipid phases encapsulating siRNA enhance lipid nanoparticle mediated transfection. Nat Commun.

[44] Chatterjee S, Kon E, Sharma P, Peer D (2024). Endosomal escape: A bottleneck for LNP-mediated therapeutics. Proc Natl Acad Sci U S A.

[45] Winkeljann B, Keul DC, Merkel OM (2023). Engineering poly- and micelleplexes for nucleic acid delivery - A reflection on their endosomal escape. J Control Release.

[46] Omo-Lamai S, Wang Y, Patel MN (2024). Lipid nanoparticle-associated inflammation is triggered by sensing of endosomal damage: engineering endosomal escape without side effects. bioRxiv.

[47] Maugeri M, Nawaz M, Papadimitriou A (2019). Linkage between endosomal escape of LNP-mRNA and loading into EVs for transport to other cells. Nat Commun.

[48] (2024). Welsh JA, Goberdhan DCI, O’Driscoll L, et al; MISEV Consortium. Minimal information for studies of extracellular vesicles (MISEV2023): from basic to advanced approaches. J Extracell Vesicles.

[49] Zhao D, Tao W, Li S (2021). Apoptotic body-mediated intercellular delivery for enhanced drug penetration and whole tumor destruction. Sci Adv.

[50] Liu Y, Hu D, Gao D (2023). Engineered apoptotic bodies hitchhiking across the blood-brain barrier achieved a combined photothermal-chemotherapeutic effect against glioma. Theranostics.

[51] Kooijmans SAA, Stremersch S, Braeckmans K (2013). Electroporation-induced siRNA precipitation obscures the efficiency of siRNA loading into extracellular vesicles. J Control Release.

[52] Rädler J, Gupta D, Zickler A, Andaloussi SE (2023). Exploiting the biogenesis of extracellular vesicles for bioengineering and therapeutic cargo loading. Mol Ther.

[53] Rezaie J, Feghhi M, Etemadi T (2022). A review on exosomes application in clinical trials: perspective, questions, and challenges. Cell Commun Signal.

[54] O'Brien K, Breyne K, Ughetto S, Laurent LC, Breakefield XO (2020). RNA delivery by extracellular vesicles in mammalian cells and its applications. Nat Rev Mol Cell Biol.

[55] Jin Y, Ma L, Zhang W, Yang W, Feng Q, Wang H (2022). Extracellular signals regulate the biogenesis of extracellular vesicles. Biol Res.

[56] Liu C, Liu D, Wang S, Gan L, Yang X, Ma C (2023). Identification of the SNARE complex that mediates the fusion of multivesicular bodies with the plasma membrane in exosome secretion. J Extracell Vesicles.

[57] Bonsergent E, Grisard E, Buchrieser J, Schwartz O, Théry C, Lavieu G (2021). Quantitative characterization of extracellular vesicle uptake and content delivery within mammalian cells. Nat Commun.

[58] Heath N, Osteikoetxea X, de Oliveria TM (2019). Endosomal escape enhancing compounds facilitate functional delivery of extracellular vesicle cargo. Nanomedicine.

[59] Brock DJ, Kustigian L, Jiang M (2018). Efficient cell delivery mediated by lipid-specific endosomal escape of supercharged branched peptides. Traffic.

[60] Shete HK, Prabhu RH, Patravale VB (2014). Endosomal escape: a bottleneck in intracellular delivery. J Nanosci Nanotechnol.

[61] Gandek TB, van der Koog L, Nagelkerke A (2023). A comparison of cellular uptake mechanisms, delivery efficacy, and intracellular fate between liposomes and extracellular vesicles. Adv Healthc Mater.

[62] Bebelman MP, Bun P, Huveneers S, van Niel G, Pegtel DM, Verweij FJ (2020). Real-time imaging of multivesicular body-plasma membrane fusion to quantify exosome release from single cells. Nat Protoc.

[63] Perrin P, Janssen L, Janssen H (2021). Retrofusion of intralumenal MVB membranes parallels viral infection and coexists with exosome release. Curr Biol.

[64] Ribovski L, Joshi BS, Gao J, Zuhorn I (2023). Breaking free: endocytosis and endosomal escape of extracellular vesicles. Extracell Vesicles Circ Nucleic Acids.

[65] Vermeulen LMP, De Smedt SC, Remaut K, Braeckmans K (2018). The proton sponge hypothesis: fable or fact?. Eur J Pharm Biopharm.

[66] Russell AE, Sneider A, Witwer KW (2019). Biological membranes in EV biogenesis, stability, uptake, and cargo transfer: an ISEV position paper arising from the ISEV membranes and EVs workshop. J Extracell Vesicles.

[67] Kopac T (2021). Protein corona, understanding the nanoparticle-protein interactions and future perspectives: a critical review. Int J Biol Macromol.

[68] Schrijver DP, de Dreu A, Hofstraat SRJ (2021). Nanoengineering apolipoprotein A1‐based immunotherapeutics. Adv Ther.

[69] Dar SA, Thakur A, Qureshi A, Kumar M (2016). siRNAmod: a database of experimentally validated chemically modified siRNAs. Sci Rep.

[70] Nie T, Heo YA, Shirley M (2023). Vutrisiran: a review in polyneuropathy of hereditary transthyretin-mediated amyloidosis. Drugs.

[71] Brown CR, Gupta S, Qin J (2020). Investigating the pharmacodynamic durability of GalNAc-siRNA conjugates. Nucleic Acids Res.

[72] Robb KP, Galipeau J, Shi Y, Schuster M, Martin I, Viswanathan S (2024). Failure to launch commercially-approved mesenchymal stromal cell therapies: what’s the path forward? Proceedings of the International Society for Cell & Gene Therapy (ISCT) Annual Meeting Roundtable held in May 2023, Palais des Congrès de Paris, Organized by the ISCT MSC Scientific Committee. Cytotherapy.

[73] Prasannan A, Debele TA, Tsai HC, Chao CC, Lin CP, Hsiue GH (2015). Synthesis and evaluation of the targeted binding of RGD-containing PEGylated-PEI/DNA polyplex micelles as radiotracers for a tumor-targeting imaging probe. RSC Adv.

[74] Hatit MZC, Lokugamage MP, Dobrowolski CN (2022). Species-dependent in vivo mRNA delivery and cellular responses to nanoparticles. Nat Nanotechnol.

